# Treatment-Induced Diabetic Neuropathy: A Case Report on Multimodal Pain Management in a Young Patient With Type I Diabetes

**DOI:** 10.7759/cureus.99918

**Published:** 2025-12-23

**Authors:** Seung J Lee, Jaimin Pandya, Kanchana Gattu, Thelma Wright

**Affiliations:** 1 Anesthesiology, University of Maryland School of Medicine, Baltimore, USA; 2 Anesthesiology, University of Maryland Medical Center, Baltimore, USA

**Keywords:** diabetic neuropathic pain, insulin pump, multi-modal pain management, neuropathic pain, treatment induced diabetic neuropathy

## Abstract

Treatment-induced diabetic neuropathy (TIND), also known as insulin neuritis, is a rare but important complication that can occur after rapid correction of chronic hyperglycemia in patients with long-standing diabetes. It manifests as acute, severe neuropathic pain with autonomic dysfunction despite improved glycemic control. Chronic neuropathic pain, which arises from peripheral or central nervous system dysfunction, includes a broad spectrum of conditions such as diabetic neuropathy, trigeminal neuralgia, spinal cord injury, post stroke pain, and radiculopathy. However, TIND remains underrecognized and frequently misdiagnosed.

This case report presents a 25-year-old male with type 1 diabetes who experienced two distinct episodes of treatment-induced diabetic neuropathy following periods of rapid glycemic improvement at a tertiary pain management center in the United States. The first episode occurred in 2019 after initiation of intensified insulin therapy, and the second in 2023 following insulin pump placement. Both episodes were characterized by severe bilateral lower extremity burning and stabbing pain despite normal imaging and electrodiagnostic studies.

His pain management required a multimodal strategy incorporating pharmacologic therapy (gabapentinoids, antidepressants, and opioids), psychological support, physical therapy, and interventional pain management with lumbar sympathetic blocks. TIND should be considered in patients with unexplained neuropathic pain following glycemic correction. A gradual approach to glycemic control may mitigate its severity. Multimodal pain management, including pharmacotherapy, physical therapy, psychological support, and interventional techniques, plays a crucial role in optimizing outcomes for these patients.

## Introduction

Neuropathic pain is defined as pain arising as a direct consequence of a lesion or disease affecting the nervous system [1]. It may result from either peripheral or central nervous system dysfunction and encompasses a wide spectrum of conditions, including diabetic neuropathy, trigeminal neuralgia, spinal cord injury, post-stroke pain, polyneuropathy, and radiculopathy [2]. Among these, diabetic neuropathy remains one of the most prevalent complications of diabetes mellitus, significantly impairing quality of life and posing therapeutic challenges [2,3].

Treatment-induced diabetic neuropathy (TIND), also known as insulin neuritis, is a rare yet distinct iatrogenic form of diabetic neuropathy that typically develops after rapid correction of hyperglycemia in diabetic patients [4]. It is characterized by the abrupt onset of severe neuropathic pain with autonomic dysfunction, occurring within weeks of achieving improved glycemic control [4]. Although uncommon, this condition can occur in both type 1 and type 2 diabetes and is often underrecognized, leading to delayed diagnosis and suboptimal management [5]. The incidence of TIND in patients with diabetes is estimated to be approximately 10% among those referred for evaluation of diabetic neuropathy at tertiary centers [6].

The pathophysiology of TIND remains unclear, but rapid glucose normalization is thought to trigger microvascular ischemia and metabolic stress to the dorsal root ganglia, resulting in small-fiber and autonomic injury [4,7]. A leading theory proposes that nerves long exposed to high glucose enter a state of metabolic shock when glucose levels fall too quickly. This abrupt mismatch between energy supply and demand can injure small, vulnerable nerve fibers, similar to an electrical grid destabilizing when power input drops abruptly. Diagnosis is clinical, supported by symptom onset shortly after glycemic improvement and typically normal EMG and laboratory studies.

This case report describes a young man with type 1 diabetes who experienced two distinct episodes of TIND. The first episode occurred in 2019, following partial glycemic correction after the initiation of intensified insulin therapy. This corresponded with worsening bilateral lower extremity burning pain, which improved after achieving stable yet elevated Hemoglobin A1c (HbA1c) levels of 11.3% in 2019, down from 15.4% in 2018. The second episode started after initiation of insulin pump therapy in May 2023, when his HbA1c decreased rapidly from 14.7% to 6.7% within seven months. Both episodes were temporally associated with significant improvements in glycemic control and acute exacerbations of neuropathic pain, highlighting the potential for recurrent TIND in patients with fluctuating or aggressively corrected glucose levels. This case underscores the importance of gradual glycemic correction and multidisciplinary management to prevent and address this debilitating complication.

This case highlights the diagnostic challenges of recurrent TIND, the potential severity of symptoms that may require a flexible multimodal management strategy, and the critical importance of gradual glycemic correction to reduce the risk of this iatrogenic complication.

The content of the case report was previously presented as a poster presentation at the American Society of Regional Anesthesia and Pain Medicine (ASRA) Pain Medicine Meeting on November 22, 2024, held in Las Vegas, Nevada.

## Case presentation

Currently, a 25-year-old male with type 1 diabetes mellitus (diagnosed at age nine), asthma, chronic pancreatitis, vitamin D deficiency, and depression was initially referred to the pain management center in 2019 by the neurology service for evaluation of bilateral lower-extremity pain. At that time, he was a college student studying film and denied tobacco, alcohol, or illicit drug use. The patient’s first episode of treatment-induced diabetic neuropathy (TIND) occurred in 2019.

The patient developed abrupt burning and stabbing pain in both legs in September 2019, described as constant and worse at night. Previously, his diabetes had been poorly controlled for years, with hemoglobin A1c levels between 13.3 % and 15.4 % between 2017 and 2018. After intensification of insulin therapy, his A1c improved modestly to 11.3 % (Table 1) in 2019, coinciding with worsening neuropathic pain in a stocking distribution extending from the feet to the thighs.

**Table 1:** Hemoglobin A1c (HbA1c) and Estimated Average Glucose (EAG) Levels (June 2017 – October 2019) Reference ranges: HbA1c 4.0–5.6%; estimated average glucose (EAG) ≈ 70–126 mg/dL; A1c-Derived Average Glucose Formula: EAG calculated as 28.7 × A1c – 46.7;↑ = Above reference range.

Date (MM/DD/YYYY)	Hemoglobin A1c (%)	Estimated Average Glucose (mg/dL)
06/09/2017	13.7 ↑	≈ 347
11/06/2017	14.5 ↑	≈ 370
01/29/2018	13.3 ↑	≈ 335
12/07/2018	15.4 ↑	≈ 395
10/15/2019	11.3 ↑	≈ 278

Extensive evaluation, including normal electromyography (EMG) and spinal MRI, negative infectious and autoimmune studies, and a skin biopsy, showed reduced intraepidermal nerve fiber density (IENFD), which was consistent with small-fiber neuropathy secondary to diabetes.

No interventional procedures were performed during this period. His blood glucose remained chronically elevated afterward, with an A1c around 11%, and the pain was functionally manageable with multimodal pharmacologic therapy and behavioral support. His initial medication regimen consisted of topiramate 50 mg twice daily, gabapentin 1200 mg three times daily, nortriptyline 50 mg at bedtime, cyclobenzaprine 5 mg three times daily as needed, and duloxetine 60 mg daily.

He also participated in physical therapy and pain psychology, with interventions centered on adaptive coping strategies, cognitive-behavioral techniques to address catastrophizing and fear-avoidance, and behavioral activation. Physical therapy incorporated graded desensitization and progressive activity pacing to restore functional mobility and support reintegration into daily and academic activities. By late 2020, his symptoms had stabilized, allowing his medication regimen to be streamlined in 2021 to pregabalin 100 mg twice daily, duloxetine 60 mg daily, and nortriptyline 50 mg at bedtime. At this stage, his symptoms were controlled without opioid or interventional therapy, and he maintained his studies and daily function.

The patient’s second episode of TIND occurred in 2023. In May 2023, the patient was transitioned to an Omnipod 5 insulin pump with continuous glucose monitoring, resulting in a rapid decline in Hb A1c from 14.7 % (May 2023) to 6.7 % (December 2023) within seven months (Table 2). Soon after this correction, he developed severe burning, stabbing, and electric-like pain radiating from the hips to the toes, accompanied by insomnia, depressed mood, and marked functional decline, consistent with a second, more severe episode of TIND.

**Table 2:** Hemoglobin A1c (HbA1c) and Estimated Average Glucose (EAG) Levels (March 2023 – December 2023) Reference ranges: HbA1c 4.0–5.6%; estimated average glucose (EAG) ≈ 70–126 mg/dL; A1c-Derived Average Glucose Formula: EAG calculated as 28.7 × A1c – 46.7; ↑ = Above reference range.

Date (MM/DD/YYYY)	Hemoglobin A1c (%)	Estimated Average Glucose (mg/dL)
03/27/2023	>15.0 ↑	>385
05/01/2023	14.7 ↑	≈ 376
12/28/2023	6.7 ↑	≈ 146

Initially, his pharmacologic regimen was pregabalin 100 mg three times daily and duloxetine 90 mg daily, with topical lidocaine 5% ointment. During the TIND episode, pregabalin was gradually titrated upward to 150 mg three times daily by November 2023 with duloxetine 90 mg daily, with topical lidocaine 5% ointment under close medical supervision. Due to disabling pain and impaired mobility, hydrocodone-acetaminophen 5/325 mg was introduced in August 2023 and titrated from 30 tablets/month to 120 tablets/month by late fall 2023 for breakthrough pain.

**Figure 1:**
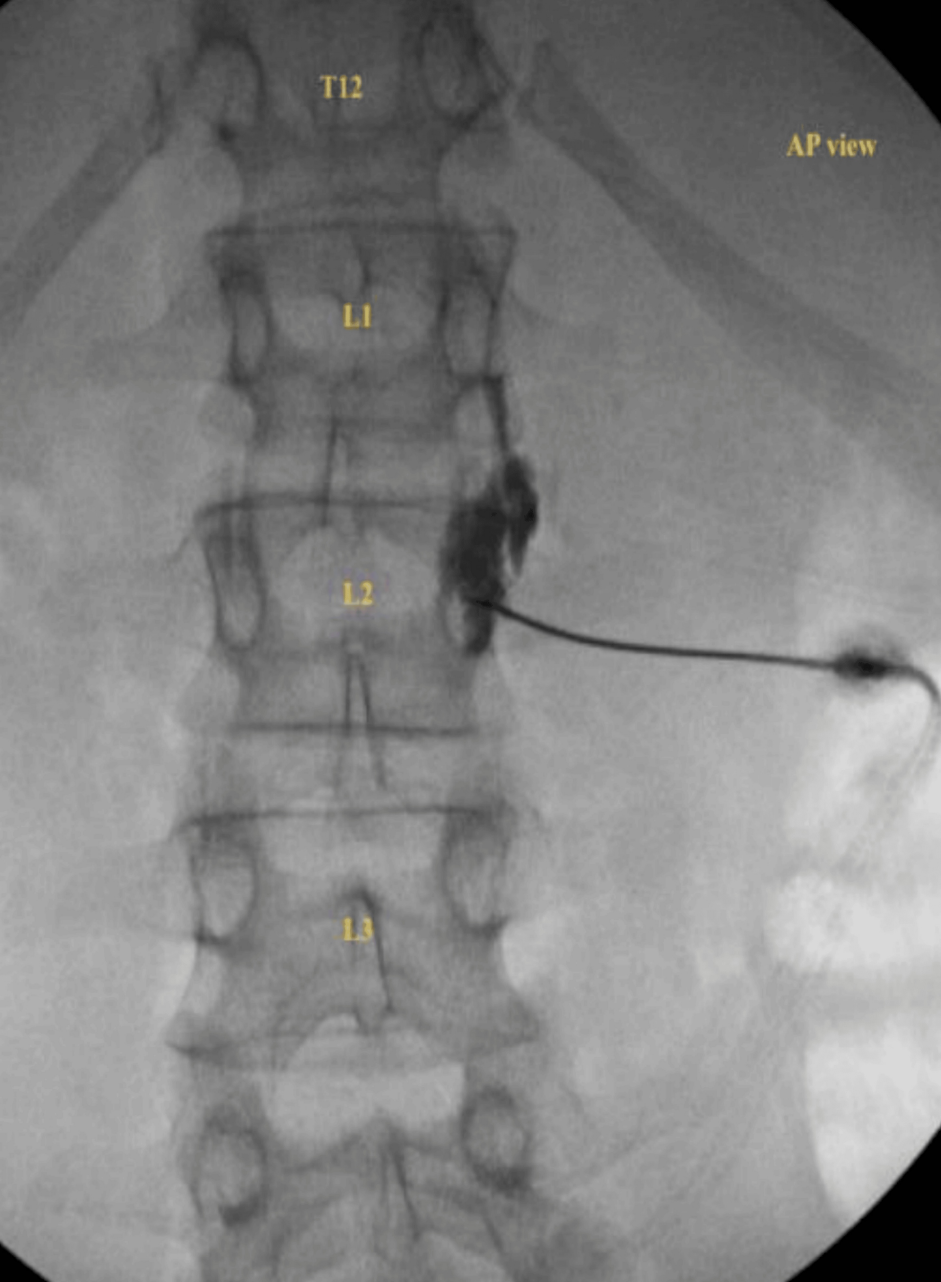
Anteroposterior Fluoroscopic View of Right Lumbar Sympathetic Block at L2 An anteroposterior fluoroscopic image demonstrating needle placement at the right L2 vertebral level.  Image obtained using standard fluoroscopy with moderate contrast and resolution settings to optimize visualization of bony landmarks and contrast spread.

**Figure 2:**
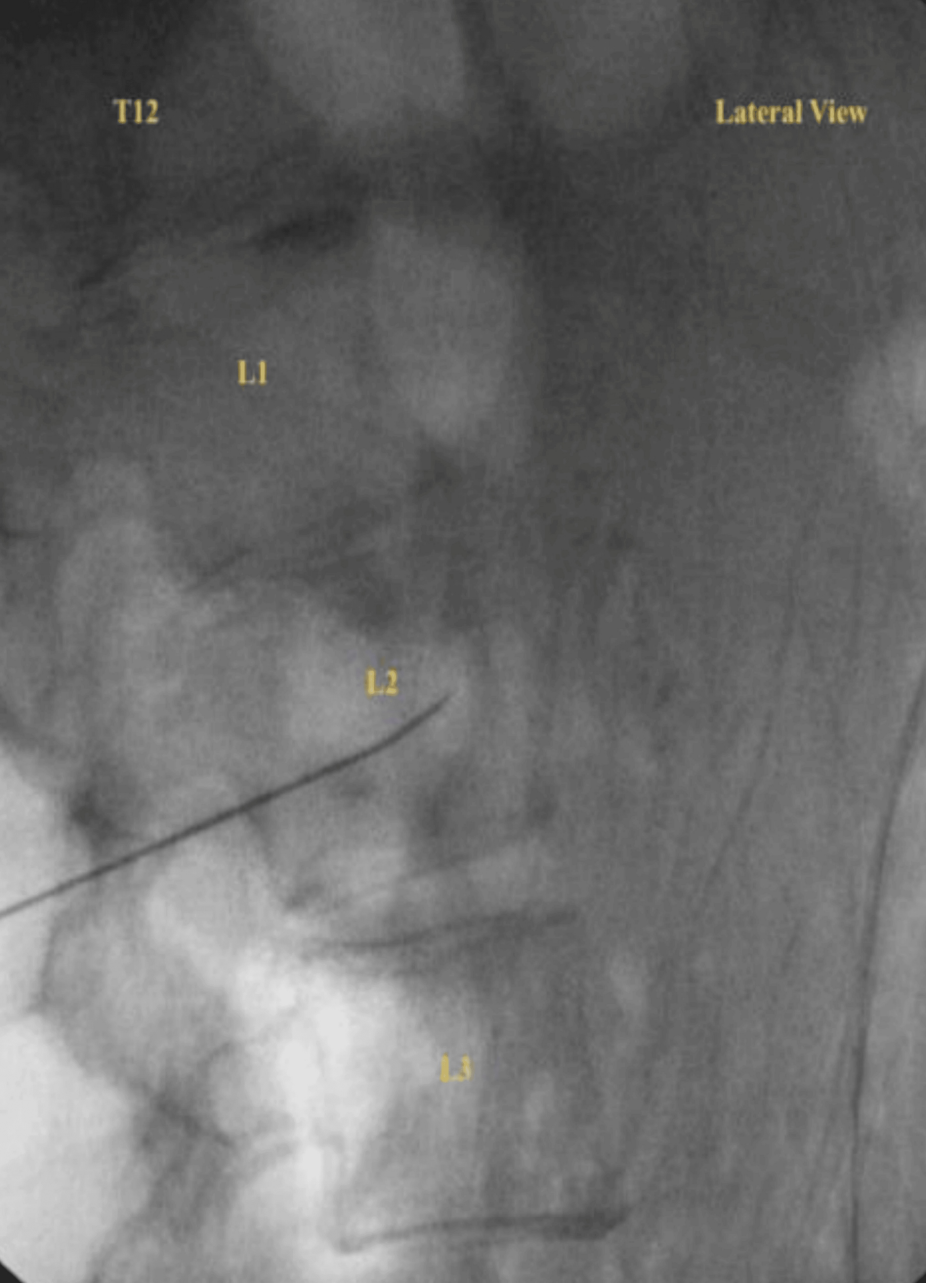
Lateral Fluoroscopic View Confirming Needle Depth at L2 A lateral fluoroscopic image showing depth confirmation of the needle tip at the right L2 level.  Image acquired using standard fluoroscopic lateral projection with adequate soft-tissue resolution for procedural confirmation.

He underwent a single right-sided lumbar sympathetic block in September 2023 for diagnostic and therapeutic purposes after a written informed consent was obtained from the patient. Under fluoroscopic guidance, the L2 vertebral level was targeted using a 5-inch, 22-gauge spinal needle. After confirming appropriate needle placement, Omnipaque contrast was injected to verify accurate spread along the sympathetic chain. A 3 mL test dose of 1% lidocaine with epinephrine (1:100,000) was then administered to exclude intravascular uptake, followed by 12 mL of 0.2% bupivacaine for the therapeutic injection (Figures 1, 2).

The patient experienced complete immediate pain relief on the right side, with pain decreasing from 10/10 to 0/10 on the Numeric Pain Rating Scale (NPRS, 0-10) post-procedure. However, within 24 hours, the pain returned with increased severity, reaching 10+/10 on the NPRS and described by the patient as “worse than before. The intensity gradually subsided over the following week, returning to his baseline level of chronic pain. Given the exacerbation, the patient declined a contralateral block, and further interventional procedures were deferred.

Following stabilization of glycemic control, continued cognitive-behavioral pain therapy, and structured medication tapering, his pain improved steadily. By January 2024, his average pain decreased from 10/10 to 2/10 on the NPRS, and he regained independence in ambulation, sleep, and academic participation. His regimen was simplified to pregabalin 150 mg three times daily and duloxetine 90 mg daily, with hydrocodone discontinued and transitioned to tramadol as needed for breakthrough pain.

The patient continues to be followed jointly by pain management, endocrinology, and psychology with sustained functional recovery and stable glycemic control.

## Discussion

Pathophysiology

Treatment-induced diabetic neuropathy (TIND), historically referred to as insulin neuritis, is an acute iatrogenic form of diabetic neuropathy that occurs following rapid improvement in glycemic control in patients with prolonged hyperglycemia [4]. It typically presents with severe burning pain, dysesthesia, and autonomic dysfunction within weeks to months of substantial reductions in blood glucose or hemoglobin A1c (HbA1c). Although uncommon, TIND is increasingly recognized as an important cause of acute neuropathic pain in patients with type 1 and type 2 diabetes [4,7].

The pathophysiology of TIND remains incompletely understood. Proposed mechanisms include ischemic injury to the vasa nervorum and metabolic stress to the dorsal-root ganglia caused by abrupt glucose normalization, resulting in oxidative stress, endoneurial hypoxia, and microvascular dysfunction [4,7]. These changes may selectively affect small unmyelinated and autonomic fibers, explaining why many patients exhibit normal findings on electromyography and nerve-conduction studies, as was seen in our patient. The skin biopsy showed reduced intraepidermal nerve fiber density (IENFD), consistent with small-fiber neuropathy; such a reduction generally worsens as the duration or severity of diabetes increases. Experimental rat models of TIND further demonstrate that abrupt HbA1c reduction can decrease nerve-conduction velocities, reduce intraepidermal nerve fiber density, and increase macrophage infiltration, suggesting an inflammatory component with ischemic injury [8]

Clinical implications

Our case is notable for demonstrating two distinct episodes of TIND separated by several years. The first episode (2019-2020) followed modest glycemic improvement after intensified insulin therapy, producing mild to moderate neuropathic pain that was manageable with non-interventional multimodal therapy. The second episode (2023) developed after the introduction of an insulin pump, when his HbA1c fell rapidly from 14.7 % to 6.7 % within seven months. This episode was far more severe, leading to functional decline and necessitating escalation of pharmacologic and interventional pain management. The recurrence pattern emphasizes that TIND can occur not only after the first major correction of hyperglycemia but also after subsequent rapid reductions in glucose levels, even in patients with a prior history of the condition.

The literature supports the relationship between the magnitude and speed of HbA1c reduction and the likelihood of developing TIND. Gibbons and Freeman reported that a decrease in HbA1c greater than two percentage points within three months substantially increases the risk of developing TIND [4]. In such patients, gradual and stepwise glycemic correction is recommended to mitigate nerve injury. Our patient’s second episode followed an HbA1c drop of 8 percentage points within seven months, well within the high-risk range described in prior studies.

Management lessons

Management of TIND remains supportive and requires a multimodal approach that combines pharmacologic, psychological, and interventional strategies [9]. First-line pharmacotherapy includes anticonvulsants such as pregabalin and gabapentin, serotonin-norepinephrine reuptake inhibitors such as duloxetine, and tricyclic antidepressants [2, 3]. Opioids may be used briefly for refractory pain, but should be tapered as symptoms improve.

In our case, initial management during the first episode relied solely on pharmacologic therapy, whereas the second episode required additional interventional measures, including lumbar sympathetic blocks to achieve partial relief.

The brief but complete analgesic response followed by a significant rebound in pain intensity after the lumbar sympathetic block mirrors paradoxical reactions occasionally observed in sympathetically driven pain disorders, including CRPS. This phenomenon may indicate a shared mechanism involving sympathetic nervous system dysregulation or a pro-nociceptive consequence of the block itself. However, the evidence for these phenomena is limited and largely anecdotal, and the underlying mechanisms remain incompletely understood. These observations suggest that sympathetic blocks should be approached cautiously in patients with suspected TIND.

Importantly, with gradual glycemic stabilization and continued multidisciplinary care, pain psychology, physical therapy, and medication optimization, our patient experienced near-complete functional recovery, allowing de-escalation to non-opioid therapy and eventual use of tramadol only as needed.

Limitations

Recognition of TIND is crucial because its presentation may be mistaken for worsening diabetic neuropathy or another new pain syndrome, potentially prompting unnecessary diagnostic testing. Identifying the temporal relationship between glycemic improvement and pain onset is key. Our case demonstrates that recurrent or biphasic TIND can occur following separate episodes of rapid glucose correction, reinforcing the need for careful coordination among neurology, endocrinology, and pain-management teams.

This report is limited by its single-patient case report, which restricts the broader applicability of the clinical observations. Objective electrophysiologic follow-up was not performed, resulting in the absence of repeat nerve-conduction data to correlate with symptom evolution. Despite these constraints, the case provides valuable insight into the presentation and recurrence of TIND in a real-world clinical setting.

## Conclusions

This case illustrates a rare example of recurrent, biphasic treatment-induced diabetic neuropathy (TIND) in a young patient with type 1 diabetes, with two distinct episodes separated by several years-the first following moderate glycemic improvement in 2019 and the second after rapid normalization of blood glucose in 2023. While TIND is recognized as an uncommon complication of rapid glycemic correction, recurrence after a prolonged interval has seldom been reported.

This case demonstrates that rapid reductions in HbA1c can precipitate acute neuropathic symptoms and that recurrence may occur with subsequent episodes of aggressive glycemic correction. While management required a multimodal strategy, the effectiveness of individual interventions varied. This supports the need for a flexible, individualized approach, with treatment de-escalated as the acute phase resolves. Recognition of the temporal relationship between glycemic improvement and neuropathic pain is essential to guide supportive treatment and to encourage gradual, measured glycemic correction in patients at risk for TIND.

Recognition of recurrent TIND expands the understanding of this underdiagnosed condition and highlights that patients who recover from an initial episode may still be vulnerable to subsequent neuropathic flares when glycemic correction occurs too rapidly. Clinicians should suspect TIND when acute neuropathic pain emerges shortly after a marked reduction in HbA1c (typically >2% within approximately three months).
